# Acaricidal efficacy of fluralaner against *Rhipicephalus microplus* ticks under laboratory and field conditions in Brazil

**DOI:** 10.1186/s13071-025-06775-2

**Published:** 2025-04-28

**Authors:** Daniel de Castro Rodrigues, Gabriel Webert Gomes, Francisca Leticia Vale, Ana Lúcia Coutinho Teixeira, Isabela Santos Silva, Haile Dean Figueiredo Chagas, Tom Strydom, Siddartha Torres, Rafael Paranhos de Mendonça, Fernando de Almeida Borges, Lívio Costa Junior, Lorena Lopes Ferreira, Welber Daniel Zanetti Lopes, Caio Monteiro

**Affiliations:** 1 Graduate Program in Animal Science-School of Veterinary and Animal Science, Federal University of Goiás-Samambaia Campus, Nova Veneza Highway, Km 8, Goiânia, GO 74690-900 Brazil; 2 MSD Animal Health, Avenida Dr. Chucri Zaidan, 246-96, 9th Floor, São Paulo, SP CEP 04583-110 Brazil; 3 Undergraduate Program in Veterinary Medicine, Federal University of Goiás-Samambaia Campus, Nova Veneza, Km 8, Goiânia, GO 74690-900 Brazil; 4 MSD Animal Health, 20 Spartan Road, Kempton Park 1619, Isando, South Africa; 5 Merck Animal Health, 2 Giralda Farms, Madison, NJ 07940 USA; 6 Federal University of Franca, Franca, São Paulo, Brazil; 7 Federal University of Mato Grosso do Sul, Av. Senador Felinto Muller, 2443, Campo Grande, MS 79070-900 Brazil; 8 CCBS Research Center, Federal University of Maranhão, Avenida dos Santos, Portugueses, no. 1966, São Luís, MA 65080-805 Brazil; 9 Department of Preventive Veterinary Medicine, School of Veterinary Medicine, Federal University of Minas Gerais, Belo Horizonte, MG Brazil; 10 Department of Biosciences and Technology, Institute of Tropical Pathology and Public Health, Federal University of Goiás, R. 235, S/N°-University East Sector, Goiânia, GO 74605-050 Brazil

**Keywords:** Cattle tick, Isoxazolines, Discriminating dose, Larval immersion test, Field trial

## Abstract

**Background:**

The first isoxazoline-based acaricide (fluralaner) for the control of *Rhipicephalus microplus* was introduced onto the market in 2022, initially in Brazil, followed by other Latin American countries*.* Therefore, it is important to establish laboratory methods to monitor the susceptibility of populations of *R. microplus* to this molecule and to determine the relationship between the results of laboratory tests and those from field trials.

**Methods:**

A larval immersion test (LIT) was performed on 18 populations of* R. microplus*. The lethal concentration 50 (concentration causing 50% mortality [LC50]) values were calculated to determine the resistance ratios (RRs) of the populations. The lethal concentration 99 (concentration causing 99% mortality [LC99]) values were calculated to determine the discriminating doses (DDs = 2 × LC99). The DDs were applied in tests with the POA (susceptible) and GYN (resistant) strains, as well as in tests with the population that presented the lowest LC50 value (population 14) and with the two populations that presented the highest LC50 value (populations 10 and 16). Finally, we performed field trials with the population that presented the lowest and two highest LC50 values.

**Results:**

In the LIT with fluralaner, the LC50 values ranged from 0.144 to 0.481 µg/mL for the 18* R. microplus* populations. The mortality rate was 100% in the tests of the DDs in the five populations tested. In the field trials, the efficacy of fluralaner was similar for the three populations of *R. microplus* tested (populations 14, 10 and 16), with therapeutic efficacy (until day 21) of 100% and persistent efficacy (between days 28 and 42) > 95%.

**Conclusion:**

We observed natural variability in the susceptibility of larvae from the different populations of *R. microplus* that had never been treated with this compound. Despite the observed variability in the in vitro results (LC50), a comparable efficacy of > 90% lasting until day 42 was observed in the field trials. Also, based on the results of the laboratory testing (LC50 and DD) and field trials, we can conclude that there was no resistance to fluralaner in the 18 studied tick populations.

**Graphical Abstract:**

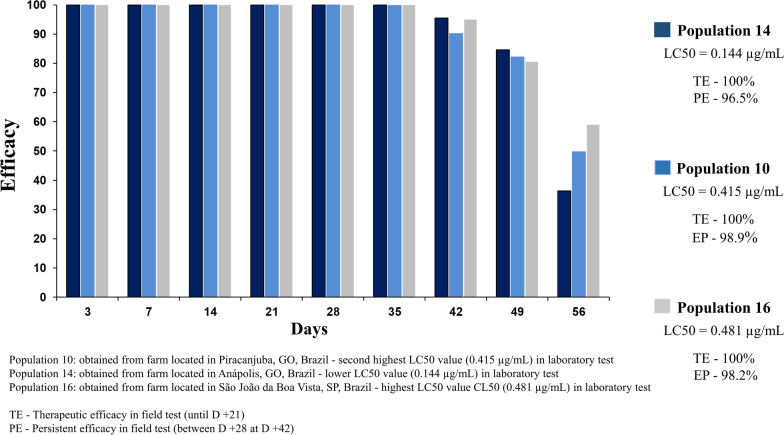

## Background

*Rhipicephalus microplus* (family Ixodidae), popularly known as the cattle tick, is an ectoparasite with a wide geographical distribution [1–3]. The economic importance of *R. microplus* is linked to its impact on meat and milk production in the cattle industry worldwide, blood loss in animals, inflammatory and allergic reactions, the transmission of pathogenic agents and expenses related to their control [4, 5]. In Brazil alone, it has been estimated that this ectoparasite is responsible for losses of 3.24 billion US dollars per year [6].

The control of *R. microplus* is based mainly on the use of acaricides, which have significantly contributed to the control of tick infestations [7–10]. However, the continuous exposure of *R. microplus* to acaricides has resulted in the selection of resistant populations in several countries around the world [8, 9, 11]. Currently, there are records of populations resistant to synthetic pyrethroids, amidines, organophosphates, macrocyclic lactones, phenylpyrazoles and benzophenylureas [8–10, 12]. In addition, climate change and global warming have resulted in an increase in the number of annual generations of *R. microplus* in certain regions, increasing the challenges associated with control of this tick [13]. These factors highlight the need for new technologies or drugs to control *R. microplus* [14, 15].

A new acaricide (Exzolt® 5%; MSD Animal Health, Rahway, NJ, USA) for tick control in cattle was introduced onto the market in 2022, initially in Brazil [16–18], followed by other Latin American countries. The active ingredient in this formulation is fluralaner, a molecule belonging to the isoxazoline class [15, 19]. Isoxazolines are considered to be the greatest innovation of this century of the antiparasitic market, with its initial introduction in 2014 to control ectoparasites in dogs [15, 19, 20].

Tests to evaluate the effect of acaricide molecules under laboratory conditions have become an important tool for understanding acaricide activity and the development of protocols for monitoring and evaluating the susceptibility of ticks to acaricides [21–23]. Information gained from monitoring efforts can contribute to strategies to delay resistance to a particular acaricide class [11]. In addition, with the establishment of laboratory study protocols, it is possible to investigate how acaricidal molecules act on different tick species, thereby clarifying the mode of action of these molecules and their effects on the morphophysiology of ticks [24–26]. Initial studies with fluralaner, under laboratory conditions, were conducted with the ticks *R. microplus*, *Rhipicephalus sanguineus* sensu lato (*R. sanguineus* s.l.) and *Ornithodoros moubata* [27, 28].

Unfed larvae and engorged females are used to evaluate acaricide activity in the laboratory, and the most commonly used techniques are the adult immersion test (AIT) [29], the larval packet test (LPT) [30] and the larval immersion test (LIT) [31, 32]. The main advantages of the tests with larvae (LPT and LIT) are: (i) large numbers of ticks can be used, which can reduce the variability in the data, consequently increasing the reliability of the results; (ii) the tests can be performed with a larger number of concentrations [23, 33, 34]. To assess the susceptibility of *R. microplus* populations to acaricides, it is recommended to perform bioassays under laboratory conditions, such as the AIT and LPT, or in field trials with animals [33, 34]. Regarding bioassays performed under laboratory conditions, LIT results have been shown to correlate well with LPT results. In addition, studies have demonstrated that the LIT can detect differences between populations with a relatively greater sensitivity [23, 34, 35]. Another advantage of using the LIT is the possibility of conducting studies based on a commercial product, which facilitates execution and reduces costs [34].

Although laboratory tests with larvae are good tools for investigating the effect of a particular molecule on ticks, there is little information regarding any correlation between the results of these laboratory tests and the results of field trials. Recently, some studies have been conducted to address this knowledge gap [36–38]. Laboratory tests can be considered to be good indicators of the susceptibility profile of a population; however, the results from efficacy field trials should be considered to be conclusive [36, 39]. Thus, it is necessary to conduct studies to establish a relationship between the results obtained under laboratory conditions and those obtained in the field, as such results will ensure more accurate laboratory tests [36–38] and also help in improving current understanding of how laboratory test results should be interpreted, thereby clarifying the strengths and limitations of the techniques. The ideal would be to carry out tests in laboratory and field conditions to definitively confirm a case of resistance that implies a decrease in the expected efficacy of an acaricidal drug under field conditions [39], especially, in a first record of resistance.

There were two objectives of the investigation reported here. First, we aimed to establish an LIT protocol to determine the acaricidal activity of fluralaner in different populations, quantify the lethal concentration 50 and 99 (concentration causing 50% and 99% mortality [LC50 and LC99], respectively) of the molecule, calculate the resistance ratio (RR) and propose and apply discriminating doses (DDs) to unfed *R. microplus* larvae. Second, we aimed to evaluate the clinical efficacy of fluralaner under field conditions, followed by establishing its correlation with the results obtained under laboratory conditions using the LIT method (i.e. LC50, RR and DD).

## Methods

### Tick populations

For the tests with larvae, we used 18 tick populations, including two reference populations: (i) the Porto Alegre (POA) strain that was susceptible to acaricides; and (ii) the GYN strain that was resistant to acaricides (synthetic pyrethroids, formadines, organophosphates and phenylpyrazoles) [38]. The GYN and POA populations were maintained through experimental infestations. The other 16 tick populations were obtained by collecting engorged females from naturally infested cattle without records of recent use of acaricides from farms located in different regions of Brazil (Table 1). All tests were carried out before Exzolt® was introduced onto the market; therefore, none of the tick populations had a history of contact with fluralaner or any other isoxazoline.

**Table 1:** Lethal concentrations of fluralaner in unfed larvae of *Rhipicephalus microplus* populations under laboratory conditions (27 ± 1 °C and relative humidity > 80 ± 5%)

Population^a^	City and State	LC50^b^	Confidence interval	RR50^c^	LC99^b^	Confidence interval	Mortality at the two concentrations used for LC50 calculations	
							1.56 µg/mL	3.12 µg/mL
POA	Porto Alegre—RS	0.208	0.203–0.213		0.777	0.720–0.846	100	100
GYN	Goiânia—GO	0.167*	0.162–0.173	0.803	0.812	0.759–0.873	100	100
1	Padre Paraíso—MG	0.156*	0.150–0.164	0.750	1.530*	1.355–1.747	99.9	100
2	Iturama—MG	0.244	0.234–0.253	1.173	1.650*	1.515–1.810	100	100
3	Planaltina—DF	0.272*	0.265–0.281	1.308	1.271*	1.192–1.362	100	100
4	Nova Friburgo—RJ	0.186*	0.180–0.191	0.894	0.736	0.684–0.799	100	100
5	Bela Vista de Goiás—GO	0.183*	0.176–0.191	0.880	1.216*	1.103–1.353	100	100
6	São Miguel do Passa Quatro—GO	0.287*	0.281–0.293	1.380	0.841	0.795–0.893	100	100
7	Uruçuca—BA	0.411*	0.400–0.424	1.976	1.547*	1.438–1.674	100	100
8	Rio Espera—MG	0.210	0.205–0.215	1.010	0.671	0.630–0.720	100	100
9	Pará de Minas—MG	0.198	0.194–0.203	0.952	0.753	0.707–0.807	100	100
10	Piracanjuba—GO	0.415*	0.402–0.428	1.995	2.352*	2.170–2.566	100	100
11	Ingaí—MG	0.292*	0.284–0.300	1.404	1.070*	0.992–1.163	100	100
12	Esmeraldas—MG	0.313*	0.302–0.324	1.505	2.523*	2.295–2.796	100	100
13	Bonfinópolis—MG	0.375*	0.361–0.390	1.803	3.371*	3.043–3.766	100	100
14	Anápolis—GO	0.144*	0.140–0.148	0.692	0.567*	0.532–0.608	100	100
15	Jataí—GO	0.216	0.207–0.225	1.038	2.197*	1.959–2.490	100	100
16	São João da Boa Vista—SP	0.481*	0.472–0.491	2.313	2.060*	1.517–1.828	100	100
		LC99 considering mortality data from all populations			1.581	1.546–1.617		

^a^POA is the strain susceptible to acaricides. GYN is the strain resistant to synthetic pyrethroids, formamidines, organophosphate and phenylpyrazoles. Populations 1—16 were obtained from samples of *R. microplus* collected on farms in different regions of Brazil

^b^LC50 and LC99 are the lethal concentrations (LC; µg/mL = ppm) producing 50% and 99% mortality, respectively, among the tested study populations. Asterisks indicate significantly different values of LC50 and LC99 relative to the POA strain

^c^RR50 is the resistance ratio, calculated from the LC50

In these experiments, we used larvae aged between 15 and 21 days after hatching that were obtained from the eggs of engorged females. To obtain the larvae, the engorged females and eggs were incubated at 27 ± 1 °C and a relative humidity (RH) > 80 ± 5% (BOD incubator SL200/364; SOLAB, Piracicaba, SP, Brazil).

### Acaricides and solvents

The solvent dimethylsulfoxide (DMSO) was purchased from Sigma‒Aldrich (St. Louis, MO, USA). A pour-on commercial formulation containing fluralaner (Exzolt® 5%, 2.5 mg/kg; MSD Animal Health) was donated by MSD Animal Health.

### LIT with different concentrations of fluralaner to determine LC50 and LC99

In this step of the experiment, we used the LIT as described by Sabatini et al. [32] with modifications. The choices of the solvent and concentrations, as well as of the test methods, were based on the descriptions of previous laboratory studies involving fluralaner and the ticks *R. microplus*, *R. sanguineus* and *O. moubata* [27, 28]. In the present study, dilutions of fluralaner were prepared from a commercial pour-on formulation (Exzolt® 5%) for the control of cattle ectoparasites.

In the LIT, approximately 500 unfed larvae were transferred with small brushes into 1.5-mL Eppendorf tubes containing the test solutions where they were immersed in the solution for 3 min. During the immersion period, the tubes were shaken vigorously, following which the solution was poured out, and approximately 100 larvae were recovered and placed in the center of a filter paper sheet (6 × 6 cm), which was folded in the middle and sealed at the ends with clips. The test concentrations were 0.024, 0.048, 0.095, 0.19, 0.29, 0.39, 0.78, 1.56 and 3.125 µg/mL (= 0.024, 0.048, 0.095, 0.19, 0.29, 0.39, 0.78, 1.56 and 3.12 ppm, respectively). Two control groups were also included, one with distilled water and the other with only the solvent 2% DMSO.

This procedure was carried out with all of the *R. microplus* populations. Five repetitions were performed for each concentration, and the experiments were repeated twice on different days, with the exception of the experiments with the GYN and POA populations, which were repeated on five different days, respectively. All sets were placed in a BOD at 27 ± 1 °C and RH > 80 ± 5% (model SL200/364; SOLAB) for 24 h, following which the mortality percentages were determined.

### LIT with DDs of fluralaner

In this step, we carried out tests with the two DDs (1.55 and 3.16 µg/mL), which had been calculated from the LC99 and established by means of probit analysis with the mortality data from the LIT with fluralaner. A control group was perfomed with DMSO 2%. The details on how the DDs were calculated are described in the Statistical Analysis section.

The LIT with the DDs was performed applying the same method as that used to determine the LC50 and LC99 values, and mortality was determined after 24 h. This test was carried out with larvae of the POA and GYN populations, with the population that had the lowest LC50 value and with two populations that had the highest LC50 values.

### Field trials with fluralaner (5% pour-on, 2.5 mg/kg)

Field trials were conducted at the same farms from which the tick populations with the lowest LC50 value (population 14: Anápolis, Girolando [GO]) and the two highest LC50 values (population 10: Piracanjuba, GO; population 16: São João da Boa Vista, São Paulo [SP]) were obtained. The experiments were performed from October 2022 to January 2023, on the Girolando breed of cattle at the three farms where the ticks were collected (farm of origin): (i) population 14, age between 5 and 6 months (females), with average weight of 156 kg (range: 137–182 kg); (ii) population 10, age between 20 and 24 months (females), with average weight of 524.85 kg (range: 435–605 kg); and (iii) population 16, age ranging from 7 to 9 months (females), with average weight of 374.95 kg (range: 298–462 kg). On these farms, the animals were kept in pastures of *Urochloa brizantha*, a common pasture grass in Brazil, and provided with a supply of corn silage along with mineral salts and water ad libitum.

The evaluations were carried out according to the standards of the Brazilian Ministry of Agriculture, Animal Husbandry and Supply (MAPA) [40] and the guidelines of the World Association for the Advancement of Veterinary Parasitology (WAAVP) [41]. At each farm, we selected 20 animals with good nutritional status and without records of treatment with acaricides in the last 90 days. Animals exhibiting natural infestation with at least 15 *R. microplus* females (length: 4.5–8 mm) on the left side of the body were included in the study. Twenty animals from each farm were divided into two experimental groups of 10 animals each, based on the average number of *R. microplus* females (length: 4.5–8.0 mm) on the left side of the animals on day -3, -2 and -1. The experimental groups were randomized according to the following criteria: after the animals were listed in decreasing order by the average number of ticks (3 counts), the two animals with the highest counts were allocated to the first repetition, the next two to the second repetition, and so on until 10 repetitions were established (10 animals per group). Then, in each block, the animals were randomly allocated to groups.

On day 0 (treatment day), the pour-on formulation of Exzolt 5%® (fluralaner 2.5 mg/kg) was applied to the back of each animal. Animals enrolled in the control group were left untreated. Each animal was weighed prior to applying the product to determine the correct volume of the product to be applied. After this treatment, the numbers of *R. microplus* females with lengths between 4.5 and 8.0 mm on days +3, +7, +14, +21, +28, +35, +42, +49 and +56 were determined. The efficacy was calculated based on the arithmetic means according to the formula recommended by Roulston et al. [42] and adopted by the MAPA [40] and WAAVP [41]:

$$\text{Efficacy percent}=\left(1-\frac{Ta x Cb}{Tb x Ca} \right)x 100$$,

where *Ta* = the mean number of female ticks counted on the treated animals after treatment; *Tb* = the mean number of ticks counted on the treated animals during the 3 days preceding the treatment date; *Ca* = the mean number of female ticks counted on the untreated control animals after the treatment date; and *Cb* = the mean number of ticks counted on the untreated control animals during the 3 days preceding the treatment date.

### Statistical analysis

The larval mortality data were used to calculate the lethal concentrations (LC50 and LC99) needed to achieve 50% and 99% larval mortality by means of probit analysis using R Studio software (version 1.2.5001–2019; R Foundation for Statistical Computing, Vienna, Austria). The LC50 values for each population were compared with the corresponding values for the susceptible strain (POA). The LC50 and LC99 of each isolate was considered significantly different from that of the POA strain only if the 95% confidence intervals did not overlap [43].

The RR was calculated by dividing the LC50 value of a population by the LC50 of the susceptible strain POA [21–23]. The DD was determined based on the value of 2× the LC99 of the susceptible population [33, 34]. Also, we calculated the LC99 using mortality data from all populations as a single sample to determine the value of the second DD (2x CL99 calculated with data from all populations).

The data on the numbers of partially engorged females from the field experiment were log-transformed (log [count + 1[) to satisfy the requirements of a normal distribution, homogeneity of variance, residual analysis and randomness of the observations. The mean values were analyzed by means of the Tukey test (*p* ≤ 0.05), employing the GLM procedure in SAS software, version 9.4 [44].

## Results

### LIT with fluralaner: determination of the LCs, RR and DD

The POA strain (susceptible) had an LC50 of 0.208 µg/mL, and the GYN strain had a significantly lower LC50 of 0.167 µg/mL. The other 16 tick populations had LC50 values ranging from 0.144 to 0.481 µg/mL. The LC50 values of six populations (GYN, 1, 4, 5, 9 and 14) were lower than those of the susceptible population (POA). Interestingly, the GYN population, which is resistant to pyrethroids, formamidines, organophosphates and phenylpyrazoles, also presented a lower LC50 value for fluralaner than did the POA population, which was susceptible to acaricides (Table 1). When the RR was calculated, values between 0.692 and 2.313 were observed. Virtually all populations had values of < 2, except for population 16 (RR = 2.313; Table 1).

The POA strain (susceptible) had an LC99 value of 0.777 µg/mL, and the GYN strain (multiresistant) had a value of 0.812 µg/mL. The other populations had LC99 values ranging from 0.567 to 3.371 µg/mL. The LC99 values of four populations (4, 8, 9 and 14) were lower than that of the POA strain (Table 1).

### LIT with DDs of fluralaner

Two DD levels were calculated: the first used the value of twofold the LC99 of the susceptible population (POA) to the acaricides (1.55 µg/mL), and the second used the value of twofold the LC99 obtained from the calculation with data from all populations as a single sample (3.16 µg/mL).

In the tests with the two calculated DDs of fluralaner, 100% mortality was observed for the five populations tested, including the susceptible strain (POA), the multidrug-resistant strain (GYN) and the three populations (14: lowest LC50 value; 10 and 16: highest LC50 value) selected for the field study (Table 2). Interestingly, the values of the DDs established from the LC99 calculations (1.55 and 3.16 µg/mL) were very close to the values of the two highest concentrations tested for the LC50 calculations (1.56 and 3.12 µg/mL). These concentrations also resulted in 100% mortality for almost all populations, with the exception of population 1, in which mortality at a concentration of 1.56 µg/mL was 99.9% (Table 1).

**Table 2:** Mortality of unfed larvae of *Rhipicephalus microplus* populations treated with a discriminating dose of fluralaner, under laboratory conditions (27 ± 1 °C and relative humidity > 80 ± 5%)

Populations^a^	Mortality (%)		
	Control^b^	DD of 1.55 µg/mL	DD of 3.16 µg/mL
POA	2.5 ± 4.3	100	100
GYN	1.2 ± 2.1	100	100
10	1.5 ± 2.5	100	100
14	0.0	100	100
16	2.4 ± 2.2	100	100

^a^POA is the strain susceptible to acaricides. GYN is the strain resistant to synthetic pyrethroids, formamidines, organophosphate and phenylpyrazoles. Population 10: second highest LC50 (0.415 µg/mL)—obtained from farm located in Piracanjuba, GO, Brazil. Population 14: lower LC50 value (0.144 µg/mL)—obtained from farm located in Anápolis, GO, Brazil. Population 16: highest LC50 value (0.481 µg/mL)—obtained from farm located in São João da Boa Vista, SP, Brazil

*DD *Discriminating dose

^b^Control group: DMSO 2%; values are presented as the mean ± standard deviation

### Field trial (Exzolt® 5%, 2.5 mg/kg)

Three farms were selected for the field studies. The first was located in the municipality of Anápolis, GO, where tick population 14 originated; this population had the lowest LC50 value in the laboratory studies (0.144 µg/mL). The second was located in the municipality of Piracanjuba, GO, the place of origin of population 10, which had the second highest LC50 value (0.415 µg/mL). The third was located in São João da Boa Vista, SP, where population 16 originated, which had the highest LC50 value (0.481 µg/mL) (Table 1). The RRs of populations 10 and 16 (those with higher LC50 values), calculated based on comparison with the population 14, were 2.88 (population 10) and 3.34 (population 16), respectively.

In the field trials conducted on the three farms, statistically significant differences (*p* ≤ 0.05) were observed in the tick count on the cattle (control and treated groups) from day +3 up to day +49. On day +56, differences (*p* ≤ 0.05) were observed only between the control and treated groups in the studies performed on the farms with populations 14 and 16. On the farm with population 14, no female ticks (size range: 4.5–8 mm) were observed on the first 5 evaluation days (days +3 to +28), whereas on the farms with populations 10 and 16, no ticks were observed on the first 6 evaluation days (day +3 to +35) (Table 3).

**Table 3:** Mean counts of *Rhipicephalus microplus* females (size range: 4.5–8 mm) in cattle treated with a pour-on formulation containing fluralaner (2.5 mg/kg), on three farms

Days post-infection	Population 10 (LC50 = 0.415 µg/mL)^a^					Population 14 (LC50 = 0.144 µg/mL)^b^					Population 16 (LC50 = 0.481 µg/mL)^c^				
	Control	Treatment	Efficacy	*p* value	CV	Control	Treatment	Efficacy	*p* value	CV	Control	Treatment	Efficacy	*p* value	CV
0 (day of infection)	95.1a	95.6a		0.9515	11.47	19.7a	19.9a		0.8923	4.9	33.7a	33.43a		0.9613	9.55
+3	99.0a	0.0b	100	< 0.0001	16.45	22.7a	0.0b	100	< 0.0001	13.38	27.0a	0.0b	100	< 0.0001	30.37
+7	102.2a	0.0b	100	< 0.0001	18.11	24.6a	0.0b	100	< 0.0001	13.21	26.9a	0.0b	100	< 0.0001	22.89
+14	89.8a	0.0b	100	< 0.0001	16.42	29.4a	0.0b	100	< 0.0001	16.48	25.3a	0.0b	100	< 0.0001	13.33
+21	90.3a	0.0b	100	< 0.0001	19.42	30.2a	0.0b	100	< 0.0001	13.4	31.8a	0.0b	100	< 0.0001	4.23
+28	93.4a	0.0b	100	< 0.0001	16.93	37.0a	0.0b	100	< 0.0001	19.97	26.8a	0.0b	100	< 0.0001	6.51
+35	78.7a	0.0b	100	< 0.0001	23.66	41.4a	0.1b	99.8	< 0.0001	19.29	31.7a	0.0b	100	< 0.0001	8.63
+42	54.9a	2.5b	95.5	< 0.0001	28.1	41.0a	4.1b	90.1	< 0.0001	35.24	27.6a	1.4b	94.9	< 0.0001	22.01
+49	45.1a	7.0b	84.6	< 0.0001	31.39	41.5a	7.5b	82.1	0.0001	32.92	24.8a	4.80b	80.5	0.0020	47.49
+56	38.3a	24.5a	36.4	0.1597	26.08	39.4a	20.0b	49.7	0.0180	28.51	30.9a	12.60b	58.9	0.0024	35.27
Therapeutic efficacy (up to day +21)			100					100					100		
Persistent efficacy (day +28 to day +42)			98.9					96.5					98.2		

Mean followed by different letters, in the same column, for the same population, are significantly different at the 5% level

*CV* Coefficient of variation

^a^Population 10: second highest LC50 (0.415 µg/mL)—obtained from farm located in Piracanjuba, GO, Brazil

^b^Population 14: lower LC50 value (0.144 µg/mL)—obtained from farm located in Anápolis, GO, Brazil

^c^Population 16: highest LC50 value CL50 (0.481 µg/mL)—obtained from farm located in São João da Boa Vista, SP, Brazil

In the three trials performed under field conditions, the efficacy was 100% up to day +28. On days +35, +42, +49 and +56, the efficacy was 99.8%, 90.1%, 82.1% and 49.7% for population 14; 100%, 95.5%, 84.6% and 36.4% for population 10; and 100%, 94.9%, 80.5% and 58.9% for population 16, respectively. We emphasize that on the three farms, the efficacy was > 98% up to day +35, > 90% up to day +42 and > 80% up to day +49 (Table 3). The therapeutic efficacy (days +3 to +21) for all three populations was 100%, while the residual efficacy (days +28 to +42) was 98.9%, 96.5% and 98.2% for populations 10, 14 and 16, respectively (Table 3).

## Discussion

In the present study, we examined the in vitro acaricide activity/field efficacy of Exzolt 5% (fluralaner) on *R. microplus* populations that had never been in contact with this isoxazoline. In addition, we also assessed an LIT method for testing isoxazolines (especially fluralaner) under laboratory conditions in which the test concentrations, immersion time and solvent were specifically defined. With this protocol, we were able to describe the behavior of these populations in relation to exposure to fluralaner, which enabled us to calculate the LC50 and LC99, and to determine the RR and DD. Finally, field efficacy was evaluated using some of these populations of *R. microplus*, and the results of these studies were compared with the RR and DDs data. The results showed that all of the studied populations were susceptible to fluralaner.

In general, test protocols involving the use of different methodologies (LPT, LIT and TIA) under laboratory conditions to monitor the activity of acaricide molecules are proposed when commercial products have been on the market for a long time [5, 8, 9], such as those for ivermectin [9, 21], fipronil [45] and fluazuron [46]. Thus, protocols are developed used tick populations under selection pressure from these molecules. In the present study, new information regarding the dose responses of different populations of *R. microplus* was generated even before the launch of products containing fluralaner/isoxazolines onto the market. This information is highly valuable and may provide a comparative basis for future studies that may be performed to monitor the susceptibility profile of populations of this arthropod to isoxazolines.

In the present study, RR values of between 0.692 and 2.313 were calculated, and only one population (population 16) had a RR value > 2 (based on comparing the LC50 of field populations with that of the POA strain). Different criteria have been proposed to evaluate the level of resistance of ticks to acaricides [5]. For example, in previous evaluations based on RR calculations, the authors of some studies considered populations to be resistant to synthetic pyrethroids when the RR was ≥ 5.0 [47, 48], while the authors of other studies with other species of ticks used values > 10 (studies with *Amblyomma*) to classify a population as resistant [49, 50]. However, in many  studies with *R. microplus*, populations were considered to be resistant to an active ingredient when: (i) the mortality values of the studied populations were significantly different from those of the susceptible strain; and (ii) the studied populations had RR values ≥ 2 [23, 36, 45]. However, in field trials performed in animals having a natural infestation with populations 14 (with a lower LC50) and 16 (with a higher LC50), fluralaner demonstrated 100% efficacy against *R. microplus* up to day 35 after treatment, a therapeutic efficacy (days 3 to 21 after treatment) of 100% and a persistent efficacy (days 28 to 42 after treatment) > 95%. In other words, populations that exhibited LC50 differences of > 3.34 in the laboratory (compare LC50 of populations 14 and 16) tests exhibited the same efficacy response in the field trials. These field efficacy results are in agreement with the data found in earlier studies conducted with fluralaner to control *R. microplus* infestations [16]*.* In these studies, which were conducted on farms in different regions of Brazil, the authors observed a therapeutic efficacy of 100% and a residual efficacy of > 90% [16]. The results obtained in the present study for tick populations that had never been exposed to fluralaner allow us to infer that the differences observed in the LC50 values for the different populations (0.144 to 0.481 µg/mL) are due to natural variability in the susceptibility of populations observed under laboratory conditions, but such variability does not lead a reduction in efficacy in the field, as evidenced by the clinical efficacy of > 95% lasting until day +42. RR values of 3.34 do not seem to be sufficient to classify a population of *R. microplus* as resistant to fluralaner.

In the present study, based on tests with different acaricide concentrations and the establishment of lethal concentrations using probit analysis, it was possible to establish the DD from 2 × the LC99 value of the susceptible strain (POA = 1.55 µg/mL), following the recommendations in the published literature [5, 22, 33, 34]. In addition, a DD calculated from 2 × the  LC99 (3.16 µg/mL) was based on the data of all the populations; this additional calculation was performed with the objective of establishing a DD that is more representative of tick species *R. microplus*. According to the Food and Agriculture Organization of the UN (FAO) and WHO, a population of a given organism can be considered resistant when the majority of individuals in that population are able to survive the application of a given drug at a concentration that is lethal for most individuals of the same species [5, 8, 33]. Therefore, determining a DD from data obtained from several populations that had never been exposed to fluralaner seems to result in a more representative value for species *R. microplus* than determining a DD from mortality data from only one population since there is natural variability among populations. We understand that this scenario, with the possibility of carrying out tests with a molecule/acaricide in diverse populations, before the launch of the product on the market, will not always be possible, but when possible, it is a strategy that deserves to be considered.

In the test with the two DDs (1.55 and 3.16 µg/mL), 100% mortality was observed for all five populations tested: POA, GYN, population 14 (lowest LC50), population 10 and population 16 (higher LC50). Furthermore, it is worth noting that the DD values are very similar to the values of the two highest concentrations used in the dose‒response tests (1.56 and 3.12 µg/mL). At these concentrations, all populations experienced 100% mortality, except for population 1 in the treatment with a concentration of 1.56 µg/mL, in which the mortality was 99%. The DDs, as already mentioned, are used to discriminate populations of susceptible and resistant ticks based on a threshold value. In general, populations with mortality rates < 95% are considered to be resistant [5, 33, 51]. Based of our results with DDs, we conclude that all of the populations tested in the present study are susceptible to fluralaner. We also observed that the results of DDs present a better correlation with the results of the field trials (populations 10, 14 and 16), which also show that these three populations are susceptible to fluralaner.

Inconsistency between laboratory results obtained using the LPT and LIT and field trial results has also been reported by other researchers [36, 38, 52–54]. In a study with macrocyclic lactones in the laboratory, an RR of 1.7 (< 2) was calculated, which would result in this population being classified as susceptible; however, in a study under field conditions, low efficacy was observed, resulting in the authors classifying this population as resistant [36]. In a study of combinations of synthetic pyrethroids and organophosphates, laboratory results revealed an RR that ranged from 14 to 105.7; however, it was possible to control *R. microplus* with high levels of efficacy using commercial formulations with combinations of molecules belonging to these two classes [38, 55]. In another study, laboratory tests were performed with four populations of *R. microplus* using cypermethrin and chlorpyrifos (LPT) and fipronil and ivermectin (LIT) [54]. In the same study, field studies were also performed with a spray formulation (cypermethrin + organophosphate), a pour-on formulation (fipronil) and an injectable formulation (ivermectin). Inconsistencies were observed in 100% of cases for the spray formulations (cypermethrin + organophosphate), 25% of the cases for fipronil and 75% of the cases for macrocyclic lactones. In this same study, laboratory (AIT) and field tests were also performed with the spray formulation (cypermethrin + chlorpyrifos) in these four populations, and the results were 100% consistent, reinforcing the recommendation that for spray formulations, AIT is the best methodology to provide practical recommendations to farmers [54]. For this reason, in our opinion, laboratory tests should be considered indicative of the susceptibility profile of a given population, while field efficacy trials should be considered conclusive, as mentioned by Torrents et al. [36] and Nava et al. [39].

Due to these inconsistencies between laboratory results with larvae (LTP and LIT) and field results, which highlight the difficulty of using laboratory data to make practical recommendations for farmers [36, 38, 52–54], new laboratory testing methodologies are being sought to assess resistance in tick populations [56]. For fluralaner, which is available in a pour-on presentation, one approach to adapt this methodology would be to determine a LC99 and perform tests with 1 × LC99, 5 × LC99 and 10 × LC99. This methodology deserves to be investigated; however, as the authors themselves point out, we emphasize that it is important that laboratory results are validated through field trials [56].

Investigations of the susceptibility/resistance of ticks to acaricides can be performed using bioassays under laboratory conditions (LIT, LIT and TIA) or through in vivo studies (stable tests and field tests) [33, 34]. In the present study, the laboratory data obtained using LIT (i.e. the RR and DD values) combined with the field efficacy results allowed us to: (i) understand fluralaner activity against *R. microplus* under laboratory and field conditions; (ii) compare the laboratory and field data, and (3) classify all 18 populations as susceptible to fluralaner. For resistance monitoring studies, and especially for the first report of a resistant population for a compound/class acaricide, we argue that it would be interesting to carry out studies under laboratory conditions (LC50, LC99, RR and/or DD) and field trials (field and/or pen study) with the aim to increase the reliability of the results and avoid false positives or negatives. This is necessary to definitively confirm a case of resistance that implies a decrease in the expected efficacy of an acaricidal drug under field conditions [39].

## Conclusions

The study established an LIT protocol for testing *R. microplus* larvae to fluralaner. Among tick populations that had never had contact with fluralaner, there is natural variability in susceptibility to this molecule. The results indicate that the difference in the RR in laboratory tests, as observed in the present study, is not a predictor of the product efficacy in the field; however, the results of the field trial and DD tests revealed a better relationship. The laboratory data obtained using LIT (i.e. the RR and DD values) combined with the field efficacy results allowed classification of all 18 populations as susceptible to fluralaner. For resistance studies, especially for the first report of resistance for a compound/class acaricide, it is necessary carry out studies under both laboratory and field conditions. The testing protocols reported here can be used to monitor the susceptibility of cattle tick populations to fluralaner and validate strategies aimed at delaying the emergence of resistant populations.

## Data Availability

No datasets were generated or analysed during the current study.
